# Identification of the anti‐mycobacterial functional properties of piperidinol derivatives

**DOI:** 10.1111/bph.13744

**Published:** 2017-03-23

**Authors:** Collette S Guy, Esther Tichauer, Gemma L Kay, Daniel J Phillips, Trisha L Bailey, James Harrison, Christopher M Furze, Andrew D Millard, Matthew I Gibson, Mark J Pallen, Elizabeth Fullam

**Affiliations:** ^1^ School of Life SciencesUniversity of WarwickCoventryUK; ^2^ Department of ChemistryUniversity of WarwickCoventryUK; ^3^ Medical SchoolUniversity of WarwickCoventryUK

## Abstract

**Background and Purpose:**

Tuberculosis (TB) remains a major global health threat and is now the leading cause of death from a single infectious agent worldwide. The current TB drug regimen is inadequate, and new anti‐tubercular agents are urgently required to be able to successfully combat the increasing prevalence of drug‐resistant TB. The purpose of this study was to investigate a piperidinol compound derivative that is highly active against the *Mycobacterium tuberculosis* bacillus.

**Experimental Approach:**

The antibacterial properties of the piperidinol compound and its corresponding bis‐Mannich base analogue were evaluated against *M. smegmatis* and Gram‐negative organisms. Cytotoxicity studies were undertaken in order to determine the selectivity index for these compounds. Spontaneous resistant mutants of *M. smegmatis* were generated against the piperidinol and corresponding bis‐Mannich base lead derivatives and whole genome sequencing employed to determine the genetic modifications that lead to selection pressure in the presence of these compounds.

**Key Results:**

The piperidinol and the bis‐Mannich base analogue were found to be selective for mycobacteria and rapidly kill this organism with a cytotoxicity selectivity index for mycobacteria of >30‐fold. Whole genome sequencing of *M. smegmatis* strains resistant to the lead compounds led to the identification of a number of single nucleotide polymorphisms indicating multiple targets.

**Conclusion and Implications:**

Our results indicate that the piperidinol moiety represents an attractive compound class in the pursuit of novel anti‐tubercular agents.

**Linked Articles:**

This article is part of a themed section on Drug Metabolism and Antibiotic Resistance in Micro‐organisms. To view the other articles in this section visit http://onlinelibrary.wiley.com/doi/10.1111/bph.v174.14/issuetoc

**Abbreviations**
CFU: colony forming units
CL_int_: intrinsic clearance
FIC: fractional inhibitory concentration
HIV: human immunodeficiency virus
LB: Luria‐Bertani
M. bovis BCG: *Mycobacterium bovis* bacillus Calmette‐Guérin
MBC: minimum bactericidal concentration
MIC: minimum inhibitory concentration
NAT: arylamine *N*‐acetyltransferase
REMA: resazurin microtiter assay
SNP: single nucleotide polymorphism
TB: tuberculosis
TE buffer: Tris‐EDTA

## Tables of Links

**Table:** 

**TARGETS**
**Enzymes**
Arylamine N‐acetyltransferase

**LIGANDS**	
Compound 1	Rifampicin
Compound 2	

These Tables list key protein targets and ligands in this article which are hyperlinked to corresponding entries in http://www.guidetopharmacology.org, the common portal for data from the IUPHAR/BPS Guide to PHARMACOLOGY (Southan *et al.*, 2016), or to the PubChem database. Links to the Guide to PHARMACOLOGY are permanently archived in the Concise Guide to PHARMACOLOGY 2015/16 (Alexander *et al.*, 2015).

## Introduction

Tuberculosis (TB), caused by the bacillus *Mycobacterium tuberculosis* (*M. tuberculosis*)*,* is a major global pathogen and has now surpassed human immunodeficiency virus (HIV) to be the leading cause of death from a single infectious agent (WHO, 2015). In 2014, 9 million new cases of active TB were reported and 1.5 million deaths resulted from TB infection, with a significant number of deaths occurring in individuals who are co‐infected with HIV and TB (Dye and Williams, 2010, WHO, 2015). The current drug regimen to treat TB is over 40 years old and is hampered by its long duration, which is typically 6–9 months in the case of drug‐sensitive strains and frequently accompanied by toxic side effects (Zumla *et al.*, 2015). A variety of reasons, including patient non‐compliance, have led to the emergence of drug‐resistant TB strains that are highly resistant to most, if not, all – in the case of totally drug‐resistant strains of TB – of the current antibiotics that are available (Udwadia *et al.*, 2012, Klopper *et al.*, 2013). Together, this highlights the urgent need for the discovery of new antibiotics that can be used to shorten treatment time, tackle the increasing problem of clinical drug resistance and be used in combination with HIV treatments. However, there has been relatively little interest in the pursuit of new therapeutic agents for TB by the pharmaceutical industry, which may be due in part to the greatest burden of disease being present in developing countries (Cole, 2014). Despite this, over the last 10 years, there has been an increase in the discovery and development of new TB drugs through either high‐throughput phenotypic screens or target‐based approaches (Christophe *et al.*, 2009, Reynolds *et al.*, 2012, Stanley *et al.*, 2012, Lechartier *et al.*, 2014). This has resulted in a promising TB drug pipeline (Zumla *et al.*, 2013, Wallis *et al.*, 2016), comprising new antibiotic agents including the accelerated approval of bedaquiline (Andries *et al.*, 2005), which is the first FDA‐approved TB drug in 40 years, and delamanid, for use in multidrug‐resistant TB (Gler *et al.*, 2012), along with new therapeutic regimens (Zumla *et al.*, 2013, Zumla *et al.*, 2015).

**Figure 1:**
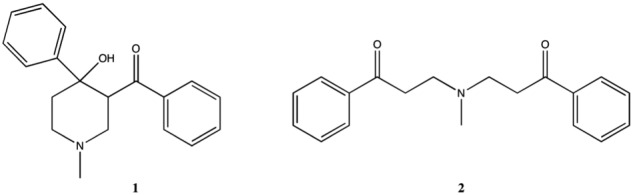
Compounds identified as inhibitors of the NAT enzyme. Structures of piperidinol compound **1** and the bis‐Mannich base compound **2.**

The mycobacterial *N*‐acetyltransferase (NAT) enzyme has been identified as a potential target for new TB agents (Bhakta *et al.*, 2004). Deletion of the *nat* gene in *M. bovis* bacillus Calmette‐Guérin (*M. bovis* BCG) results in a significant decrease in the mycolates found in the mycobacterial cell wall, with increased antibiotic susceptibility and killing within macrophages (Bhakta *et al.*, 2004, Anderton *et al.*, 2006). In previous work, a high‐throughput target‐based screen of 5000 drug‐like compounds was undertaken to find specific inhibitors of NAT enzymes (Westwood *et al.*, 2010, Fullam *et al.*, 2011, Abuhammad *et al.*, 2012, Fullam *et al.*, 2013). From this screen, the piperidinol derivative: 3‐benzoyl‐4‐phenyl‐1‐methylpiperidinol (Figure 1), compound **1**, was identified as an inhibitor of mycobacterial NAT enzymes and a potent inhibitor of *M. tuberculosis* (Fullam *et al.*, 2008, Fullam *et al.*, 2009, Westwood *et al.*, 2010, Abuhammad *et al.*, 2012, Abuhammad *et al.*, 2014). The unique *in vitro* mechanism of inhibition of compound **1** with the NAT enzyme from M. marinum has been elucidated and involves a specific covalent modification of a cysteine residue located within the active site of the NAT enzyme (Fullam *et al.*, 2008, Fullam *et al.*, 2009, Abuhammad *et al.*, 2010, Abuhammad *et al.*, 2012).

The aim of this study was to further evaluate the antimicrobial properties of the lead cyclic piperidinol derivative **1** along with its analogous bis‐Mannich base derivative **2** (Figure 1), which, depending on the *in vivo* physiological conditions encountered, may be produced via a retro‐aldol mechanism from compound **1**. To achieve this aim we undertook a combination of approaches involving antimicrobial susceptibility testing, cytotoxicity studies, resistance mapping by whole genome sequencing and genetic validation using the non‐pathogenic organism *M. smegmatis*. The combined results demonstrate that the piperidinol scaffold **1** and its derivative **2** are specific for the *Mycobacterium* genus, resulting in rapid killing, and have an encouraging cytotoxicity selectivity index with multiple and diverse mechanisms of action.

## Methods

### Chemical synthesis of 4‐hydroxy‐1‐methyl‐4‐phenylpiperidin‐3‐yl)(phenyl)methanone: compound **1**, and 3,3′‐(methylazanediyl)bis(1‐phenylpropan‐1‐one): compound **2**

The synthesis of 4‐hydroxy‐1‐methyl‐4‐phenylpiperidin‐3‐yl)(phenyl)methanone (compound **1**) and 3,3′‐(methylazanediyl)bis(1‐phenylpropan‐1‐one) (compound **2**) was carried out as described previously (Blicke and Burckhalter, 1942, Wang *et al.*, 2000, Gul *et al.*, 2002, Abuhammad *et al.*, 2012). Full experimental details and characterization data of the compounds used in this study are in Supporting Information Figures S1 and S2, [Link].

### Bacterial strains, cell lines and culture conditions

*M. smegmatis* MC^2^155 was routinely grown at 37°C in 7H9 broth (BD Difco, Oxford, UK) supplemented with 0.2% glycerol and 0.05% Tween 80 or Luria‐Bertani (LB) medium supplemented with 0.05% Tween 80. Escherichia coli (E. coli) and *Pseudomonas putida* (*P. putida*) were routinely cultured in LB medium at 37°C and 30°C respectively. Human alveolar basal epithelial A549 cells (Public Health England, Porton Down, UK, ECACC 86012804) were cultured at 37°C with 5% CO_2_ atmosphere in Ham's F‐12K (Kaighn's) Medium (Gibco, Fisher Scientific, Loughborough, UK) supplemented with 10% fetal bovine serum, 100 units·mL^−1^ penicillin, 100μg·mL^−1^ streptomycin and 250 ng·mL^−1^ amphotericin B (GE Healthcare Hyclone, Little Chalfont, UK). Ovine red blood cells were purchased from TCS Biosciences, Buckingham, UK.

### Determination of MIC

The measurement of the minimum inhibitory concentration (MIC) of compounds **1** and **2** was determined using the resazurin reduction microplate assay (REMA) as described previously (Palomino *et al.*, 2002). *M. smegmatis*, E. coli and *P. putida* were grown to mid‐log phase, and the inoculum was standardized to 1 × 10^6^ colony forming units (CFU)·mL^−1^ before addition to the prepared 96‐well flat‐bottom microtiter plate with twofold serial dilutions of each drug in media. An antibiotic control was also added to each plate (rifampicin for *M. smegmatis,* ampicillin for E. coli and tetracycline for *P. putida*), and the last column of the plate was used as a control without the addition of compound. In the case of *M. smegmatis*, the plates were incubated for 24 h at 37°C without shaking before an addition of 25 μL resazurin [one tablet of resazurin (VWR) dissolved in 30 mL of sterile PBS]. Following a further incubation at 37°C, the plates were assessed for colour development The MIC values were determined as the lowest concentration of drug that prevented the colour change of resazurin (blue – no bacterial growth) to resorufin (pink – bacterial growth). The MIC values were determined with five independent experimental repeats, and data are presented as mean ± SEM.

### Determination of the MBC

The minimum bactericidal activity (MBC) was determined by setting up a microtiter plate as performed for the MIC determination. Instead of adding resazurin at 24 h, each well from the microtiter plate was plated on to solid LB medium and the CFUs were determined after a 3 to 4 day incubation at 37°C. The lowest concentration at which no CFUs were counted was taken as the MBC. The MBCs were carried out with five independent experimental repeats.

### Time kill studies

Cultures of *M. smegmatis* (10^5^ CFU) were incubated with 2× MBC of compound **1** (625 μg·mL^−1^), 2× MBC compound **2** (312.5 μg·mL^−1^) and 2× MBC rifampicin (25 μg·mL^−1^), along with a no‐drug control. Cultures were collected at defined time intervals (0, 30, 60, 90, 120, 180, 240, 300 and 360 min and 24 h) and plated on solid LB media and incubated at 37°C for 3 days. Cell viability was assessed by determining the CFUs. The time kill studies were carried out with five independent experimental repeats, and data are presented as mean ± SEM.

### Determination of compound interactions using a REMA checkerboard assay

In order to determine whether the compounds interacted with isoniazid, we employed a checkerboard assay. Fractional inhibitory concentrations (FICs) were calculated by use of the following formula: FIC (*X* + *Y*) = [MIC of compound *X* in combination with *Y*]/[MIC of *X* alone]. To evaluate interaction profiles, we calculated the fractional inhibitory index (ΣFIC) as FIC of compound *X* + FIC of compound *Y*, where ΣFICs of ≤0.5 designate synergistic activity, ΣFICs of ≥4.0 indicate antagonism and values in between correspond to additivity (Rand *et al.*, 1993, Lechartier *et al.*, 2012).

### Cytotoxicity assay

The cytotoxicity of the compounds was measured against a human lung epithelial cell line (A549). Briefly, cells were incubated (10^6^ cells per well) with twofold serial dilutions of compounds (compound **1**: 31.25–500 μg·mL^−1^, and compound **2**: 62.5–1000 μg·mL^−1^) in a 12‐well plate (Thermo Fisher, #150628), including a cell‐only control. Following incubation for 24 h at 37°C, in the presence of 5% CO_2_, we determined cell viability by adding 100 μL resazurin for 1 h at 37°C and measuring the absorbance of the resofurin metabolite at 570 nm using a BioTek Synergy HT microplate reader. All assays were undertaken with five independent experimental repeats, and data are presented as mean ± SEM.

### Haemolysis assay

The haemolysis activity of compounds **1** and **2** was tested against ovine red blood cells. Stock concentrations of compounds **1** (500 μg·mL^−1^) and **2** (1 mg·mL^−1^) were prepared in PBS containing 5% DMSO. The compounds were serially diluted, giving final concentrations of compound **1** of 15.6–250 μg·mL^−1^ and compound **2** of 31.25–500 μg·mL^−1^. A positive control comprised lysis buffer (10 mM Tris, pH 7.8, 0.32 M sucrose, 5 mM MgCl_2_, 10% Triton X‐100), and the negative control comprised PBS containing 5% DMSO. Samples (150 μL) of each compound were added to 150 μL of ovine blood and incubated at 22°C for 1 h, after which the samples were centrifuged for 5 min (400 × *g*, 22°C). The supernatant (10 μL) was added to 90 μL of PBS in a 96‐well flat‐bottom microtiter plate (Thermo Fisher, #167008) and the absorbance read at 450 nm (BioTek HT Synergy). All assays were undertaken with five independent experimental repeats, and data are presented as mean ± SEM.

### Agglutination assay

The agglutination activity of compounds **1** and **2** was tested against ovine red blood cells. Briefly, 150 μL of ovine blood was incubated with 150 μL compound **1** or **2** (twofold dilutions: final concentration of compound **1** 15.6–250 μg·mL^−1^ and final concentration of compound **2** 31.25–500 μg·mL^−1^). Separately, 150 μL of 25% polyethylenimine was added as a positive control or 150 μL PBS as a negative control. Following addition of the compounds, we incubated the ovine blood at room temperature for 1 h, after which 50 μL of the sample was added to a round‐bottom 96‐well microtiter plate (Corning, #3790), which was then incubated at room temperature for a further 30 min. The plate was then assessed for signs of agglutination. All assays were undertaken with five independent experimental repeats, and data are presented as mean ± SEM.

### Metabolic stability in vitro

The intrinsic clearance (CL_int_) of compounds **1** and **2** was determined using mouse (CD‐1) liver microsomes (Gibco). Briefly, microsomes (100 μg final protein concentration) and test compound in 0.1 M phosphate buffer, pH 7.4, were prepared. In parallel, an NADPH‐regenerating system (Promega) was prepared in 0.1 M phosphate buffer (pH 7.4). The solutions were pre‐incubated at 37°C for 10 min before assessment of the CL_int_ was initiated by mixing the two solutions (50 μL of each; final compound concentration 1 μg·mL) at 37°C. After 0, 5, 10, 15, 20 and 30 min, the reactions were terminated by the addition of 100 μL of acetonitrile containing 1 μg·mL^−1^ verapamil and placed on ice for 30 min. The samples were then centrifuged at 12 000× *g* for 10 min and analysed by LC–MS to determine the quantity of the parent compound remaining over time. Carbamazepine (1 μg·mL^−1^) was used as a control for low CL_int_.

### Generation of spontaneous resistant mutants of M. smegmatis to compounds **1** and **2**

*M. smegmatis* resistant mutants were generated by plating 10^8^ mid‐log cells (OD_600_ 0.6) on LB agar containing 2.5× MIC of compound **1** and 5× MIC of compound **2**. In the case of compound **1**, following the initial identification of mutants from the initial 2.5× MIC plate, we inoculated the selected colonies into liquid LB media containing 2× MIC compound **1** twice before selecting on solid medium containing 2.5× MIC compound **1** and then proceeding as for compound **2**. In the case of compound **2**, following identification, we subsequently inoculated resistant mutants into liquid media in the absence of the compound to mid‐log phase and selected on solid LB agar containing 5× MIC of compound **2**. The colonies were subsequently inoculated into LB medium in the absence of a compound before selecting on LB agar plate containing either 2.5× MIC of compound **1** or 5× MIC of compound **2** to confirm resistance to the compound. Following this validation step for the generation and identification of spontaneous mutants, we inoculated the mutant strains and wild‐type *M. smegmatis* in parallel into 50 mL liquid media containing 2× MIC of the respective compound (no compound was added in the case of the wild‐type *M. smegmatis* strain) and grew to mid‐log phase. Genomic DNA was prepared (total of nine samples: seven resistant mutant strains and two wild‐type strains) by centrifugation of the culture and resuspension in 25% sucrose, 50 mM EDTA, 50 mM Tris‐HCl, pH 8.0 (SET solution), and 50 μL of 20 mg·mL^−1^ lysozyme and incubated at 37°C for 16 h. After this incubation step, 5 μL of 10 mg·mL^−1^ RNase A was added and the resuspension incubated at 37°C for 30 min, after which 250 μL of Proteinase K solution (400 mg·mL^−1^, 100 mM Tris‐HCl, pH 8.0, 0.5% SDS) was added and incubated at 55°C for 2 h. DNA was extracted using phenol–chloroform–isoamyl alcohol (24:24:1) and chloroform–isoamyl alcohol (24:1), precipitated with ethanol, dried by air and resuspended in TE buffer (10 mM Tris, 1 mM EDTA, pH 8.0). The amount and purity of the DNA were checked with a NanoDrop (Thermo Scientific).

Genomic DNA from each *M. smegmatis* mutant and wild‐type strain was converted into Nextera XT libraries for sequencing according to the manufacturer's instructions with a few modifications. Libraries were prepared using the dual indexing principle and amplified libraries were purified using 25 μL AMPure XP beads and 45 μL of purified library, retained and stored at −20°C until denaturing for sequencing. Libraries were pooled in equimolar concentrations (determined by analysis on Agilent Bioanalyser 2100 and HS dsDNA qubit assay) and 12 pM sequenced on an Illumina MiSeq platform V2 2× 250 BP paired end protocol. Reads were aligned to the *M. smegmatis* MC^2^155 (accession number NC_008596.1) using the Burrows–Wheeler Aligner MEM algorithm v0.7.5a‐r405 (Li and Durbin, 2009). Resulting BAM files were manipulated with SAMtools (Li *et al.*, 2009) to produce mpileup files, with the following parameters ‘mpileup ‐B ‐f ’.VarScan v2.3 (Koboldt *et al.*, 2012) was used for single nucleotide polymorphism (SNP) and insertion/deletion calling, with the following parameters ‘ ‐‐min‐var‐freq .80 ‐‐*P*‐value 0.05 ‐‐min‐avg‐qual 30’. SNPs that were common to both the wild‐type control and the mutant strain were not considered in further analysis. Read data have been submitted to the EBI under the accession number PRJEB15140.

### Genetic validation

Genes identified as putative targets from the whole genome sequencing data were cloned into the replicative vector pMV261 for overexpression in mycobacteria under control of the *hsp60* promoter. The gene of interest was amplified by PCR (Q5 High‐Fidelity Polymerase;New England Biolabs) from *M. smegmatis* genomic DNA. The primers used are as shown in Supporting Information Table S1. The resulting PCR product was digested with the appropriate restriction enzymes and ligated (T4 DNA ligase, NEB) into the pMV261 vector. The resulting construct was transformed into E. coli TOP10 (Invitrogen) and verified by sequencing (GATC Biotech). The resulting constructs were electroporated into *M. smegmatis* along with an empty pMV261 vector control.

### Data and statistical analysis

The data and statistical analysis in this study comply with the recommendations on experimental design and analysis in pharmacology (Curtis *et al.*, 2015). Blinding and randomization were not applied in these experiments. All data are shown as mean ± SEM of at least five individual experiments and analysed using GraphPad Prism version 7.0a software program (GraphPad Software Inc., San Diego, CA, USA). Statistical significance between different groups was determined by unpaired *t*‐test for two groups or one‐way ANOVA with Bonferroni's *post hoc* test to compare all pairs of columns for more than two groups. A value of *P* < 0.01 was considered statistically significant.

### Materials

Rifampicin was supplied by Carbosynth (Compton, UK); ampicillin, carbamazepine, isoniazid, tetracycline and verapamil, and all other chemicals were from Sigma Aldrich (Poole, UK)

## Results

### Antimicrobial properties of compound **1** and **2**

Compound **1** has previously been shown to be highly active against *M. tuberculosis* with an MIC of <5 μg·mL^−1^ (Jeney and Zsolnai, 1956, Abuhammad *et al.*, 2012). We used the REMA to determine the MIC for compound **1** and its bis‐Mannich base derivative **2** (Figure 1) against *M. smegmatis*. In addition, in order to determine the specificity of compounds **1** and **2** for the *Mycobacterium genus*, we also tested these compounds for their potential to inhibit the growth of the Gram‐negative organisms E. coli and *P. putida*. The MIC values for compounds **1** and **2** are shown in Table 1 and indicate that the piperidinol class of compounds and its corresponding bis‐Mannich base are able to inhibit the growth of *M. smegmatis* with MIC values of 62.5 and 125 μg·mL^−1^ respectively. Whilst the MIC for compounds **1** and **2** is higher than that for *M. tuberculosis*, it is clear that these compounds do not have any effect on the growth of E. coli or *P. putida* at concentrations as high as 250 μg·mL^−1^.

**Table 1:** MICs of compounds 1 and 2

Bacterium	Compound **1** **MIC (μg·mL^−1^)**	Compound **2** **MIC (μg·mL^−1^)**	Reference
*M. tuberculosis*	5	nd	(Jeney and Zsolnai, 1956; Abuhammad *et al.*, 2012)
M. bovis BCG	6.25–12.5	nd	(Abuhammad *et al.*, 2014)
*M. smegmatis*	62.5 ± 0	125 ± 0	This study
E. coli	250 ± 0	250 ± 0	This study
*P. putida*	250 ± 0	>500 ± 0	This study

nd, not determined.

All assays in this study were undertaken with *n* = 5, and data are presented as mean ± SEM.

To further understand the inhibitory properties of compounds **1** and **2**, we determined the MBCs against *M. smegmatis.* The MBCs were found to be 312.5 μg·mL^−1^ for compound **1** and ranged between 78 and 156 μg·mL^−1^ for compound **2**, indicating that both compounds are bactericidal against actively growing *M. smegmatis*. There is good correlation between the MIC and MBC values for compound **2**, whereas compound **1** displayed an MBC 5× greater than its MIC value.

**Figure 2:**
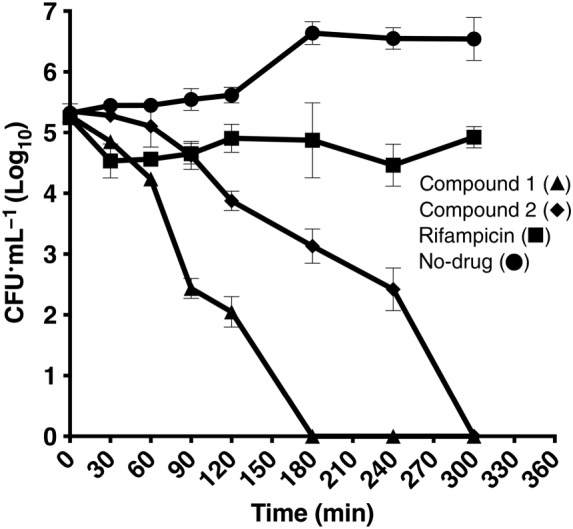
Time kill curve of compounds 1 and 2. Exponentially growing cultures of *M. smegmatis* were treated with 2x MBC of compound **1**, **2** and rifampicin. CFUs were counted at the time points indicated. The averages of five independent experiments are shown and the data are presented as mean ± SEM.

To determine whether compounds **1** and **2** are able to kill *M. smegmatis* completely and the rate at which the antimicrobial activity is exerted, we conducted time kill experiments in which the reduction of CFUs was quantified as a function of exposure time of *M. smegmatis* to 2× MBC of compounds **1** and **2** over a 24 h time period. The kill curve is shown in Figure 2. A monophasic kill curve was observed for both compounds **1** and **2** with a two‐log reduction in CFUs after 90 min for compound **1** and 3 h for compound **2** with apparently complete eradication of viable *M. smegmatis* within 3 h for compound **1** and within 5 h for compound **2**. In comparison, rifampicin was added at 2× MBC, and no reduction in CFUs were observed during this time (Figure 2) (one‐way ANOVA ).

### Compound interaction assessed by a checkerboard assay

To evaluate the impact of potential compound combinations and interactions on *M. smegmatis*, we used a checkerboard assay. Isoniazid was serially diluted twofold horizontally across the plate (0–100 μg·mL^−1^), and either compound **1** or **2** (0–1000 μg·mL^−1^) was serially diluted twofold vertically down the plate. We also used control wells in which isoniazid, compound **1** or compound **2** alone were tested (Supporting Information Figure S3, [Link]). The sum of the fractional inhibitory index (ΣFIC) for each combination was calculated, whereby ΣFIC ≤0.5 denotes synergy and ΣFIC ≥4 denotes antagonism. Compound **1** has a ΣFIC of 1.5 and compound **2** has a ΣFIC of 3. Importantly, no antagonistic interaction is observed between either compound **1** or compound **2** and the anti‐tubercular agent isoniazid.

### Cytotoxicity of compounds **1** and **2**

**Figure 3:**
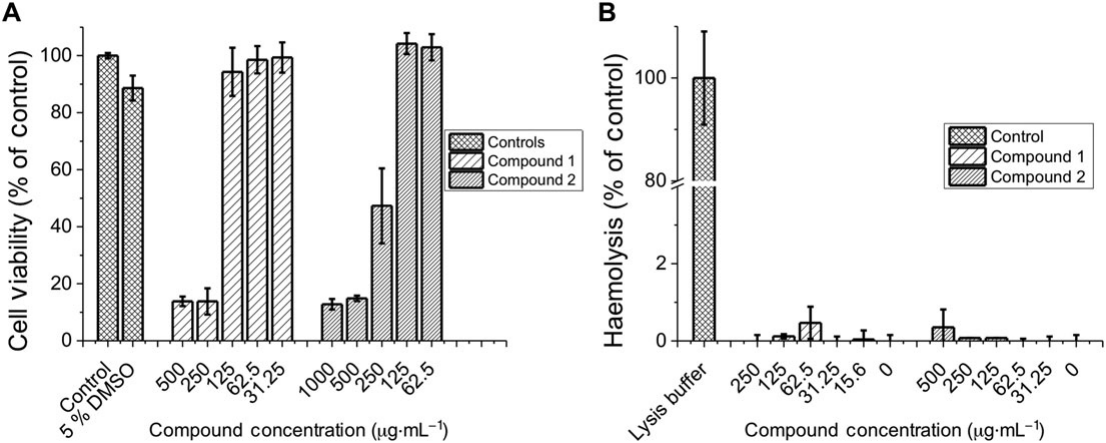
Cytotoxicity testing of compounds **1** and **2** against human A549 human lung epithelium cell line and ovine blood. (A) Effect on the cell viability of the human A549 cell line in the presence of compound **1** or **2**. A549 cells were exposed to compound **1** or **2** at the final concentrations indicated for 24 h, and the cell viability was determined after this time by the addition of resazurin. Percentage cell viability is compared with an A549 cell‐only control. (B) Haemolysis of ovine red blood cells in the presence of compound **1** or **2**. Ovine red blood cells were exposed to compound **1** or **2** at the final concentrations indicated for 1 h after which the percentage haemolysis was determined by measuring the absorbance at 450 nm. Percentage lysis is compared with the 100% lysis after addition of lysis buffer (10 mM Tris, pH 7.8, 0.32 M sucrose, 5 mM MgCl_2_, 10% Triton X‐100). In both cases, the averages of five independent experiments are shown, and the data are presented as mean ± SEM.

To determine the selectivity index of compounds **1** and **2**, we measured the cytotoxicity of both compounds against the A549 human lung epithelium cell line using the resazurin reduction viability assay and the haemolytic activity of compounds **1** and **2** against ovine red blood cells using both a haemoglobin release assay and an agglutination assay (Figure 3 and Supporting Information Figure S4, [Link]). The maximum tolerated dose of compounds **1** and **2** against A549 cells was found to be between 125 and 250 μg·mL^−1^ (*t‐*test) (Figure 3A). No lysis or agglutination of ovine blood red cells was observed at any of the concentrations tested up to 250 μg·mL^−1^ for compound **1** (*t‐*test) and up to 500 μg·mL^−1^ for compound **2** (*t‐*test) (Figure 3B and Supporting Information Figure S4, [Link]). These results therefore indicate that compounds **1** and **2** have a higher selectivity for the clinically relevant pathogenic organism *M. tuberculosis* compared with the human and ovine cells tested with a selectivity index of >30‐fold. These results are in agreement with previous studies that also found low toxicity of compound **1** for the mouse macrophage cell line RAW 264.7 and the human cell line U937 (Abuhammad *et al.*, 2012).

### Metabolic stability of compounds **1** and **2**

We subsequently evaluated the *in vitro* metabolic stabilities of compounds **1** and **2** using mouse liver microsomes and LC–MS and found that both compounds had high CL_int_ rates >48 μL·min^−1^·mg^−1^ protein. A substrate known to have a slow [CL_int_], carbamazepine, was used for comparison (Supporting Information Figure S5, [Link]).

### Target identification of compounds **1** and **2**

Compound **1** has shown encouraging inhibitory potency against *M. tuberculosis*, and biochemical studies have shown that compound **1** inhibits mycobacterial NAT enzymes (Jeney and Zsolnai, 1956, Abuhammad *et al.*, 2012, Abuhammad *et al.*, 2014). In order to identify any additional intracellular targets for the piperidinol class of compounds, we generated spontaneous resistant mutants in *M. smegmatis*. Using the determined MIC values of compounds **1** and **2** (Table 1), we generated *M. smegmatis* resistant mutants on solid LB agar containing 2.5× MIC of compound **1** (due to solubility issues) and 5× MIC compound **2**. Three colonies for compound **1** and four colonies for compound **2** were selected for subsequent inoculation in liquid medium in the absence of any compound and reselection on solid medium in the presence of either compound **1** at 2.5× MIC or compound **2** at 5× MIC. All colonies selected from the original plate maintained their ability to grow on solid medium with the addition of compounds **1** and **2** following this procedure. Two of the resistant mutants to compound **1** and two of the resistant mutants to compound **2** were retested in the REMA. The results showed that the MIC of the mutants to compound **1** increased fivefold (*t‐*test) and, to compound **2**, the MIC of the mutants increased two‐ to threefold (*t‐*test), compared with the wild‐type *M. smegmatis* strain (Table 2). Interestingly, isolate 3 to compound **1** also had increased resistance to compound **2** twofold to threefold (*t‐*test), and similarly, isolate 2 to compound **2** also increased its resistance to compound **1** between twofold and threefold (*t‐*test), indicating cross‐resistance and a potential common resistance mechanism. Each mutant remained fully susceptible to isoniazid and rifampicin (Table 2).

**Table 2:** Activity of compounds 1 and 2 against selected resistant mutants

	Isolate^a^	MIC compound **1** **(μg·mL^−1^)**	MIC compound **2** **(μg·mL^−1^)**	MIC INH (μg·mL^−1^)	MIC RIF (μg·mL^−1^)
Compound 1	1	375.0 ± 62.5	156 ± 0	25 ± 0	5 ± 0
Compound 1	3	375.0 ± 62.5	375.0 ± 62.5	12.5 ± 0	10 ± 0
Compound 2	1	171.6 ± 38.2	375.0 ± 62.5	12.5 ± 0	10 ± 0
Compound 2	2	265.6 ± 46.9	281.3 ± 31.3	12.5 ± 0	3.2 ± 0.5
n/a	*M. smegmatis* WT	56.2 ± 6.3	112.5 ± 12.5	12.5 ± 0	3.2 ± 0.5

INH, isoniazid; RIF, rifampicin; WT, wild‐type strain; n/a, not applicable.

The averages of five independent experiments are shown, and the data are presented as mean ± SEM.

a Isolate refers to mutant numbers listed in Table 3.

Given that resistance to compounds **1** and **2** was maintained in the absence of any selection pressure on the mutant strains generated, it was postulated that a genetic resistance mechanism was involved in the observed resistant phenotype. To identify the genetic mutation causing the observed phenotype, we subjected the genomes of the mutant strains to whole genome sequencing using Illumina technology (Table 3).

**Table 3:** SNPs detected in *M. smegmatis* mutants resistant to compounds 1 and 2

SNP locus *M. smegmatis chromosome position*	Codon Change	Amino acid change	Gene	Annotation	Compound 1			Compound 2			
2223880	cCc ➔ cGc	P380R	*MSMEG_2148*	HNH endonuclease	–	–	–	–	R	R	R
2223882	Aaa ➔ Caa	K381Q			–	–	–	–	K	K	K
2816638	gGa ➔ gAa	G332E	*MSMEG_2746*	Unknown	E	E	–	–	E	–	–
3105561	Ggg ➔ Agg	G278R	*MSMEG_3033*	*aroB*	–	–	–	R	R	R	R
3105568	gGc ➔ gAc	G280D			–	–	–	–	D	D	D
3105783	Tgc ➔ Ggc	C352G			–	–	–	G	G	G	G
3105805	cTa ➔ cCa	L359P			–	–	–	–	P	P	P
3328246^a^	ccT ➔ ccC	P384P	*MSMEG_3244*	Hypothetical	–	–	–	–	P^a^	P^a^	P^a^
4158542^a^	ttT ➔ ttC	F227F	*MSMEG_4083*	Monooxygenase	–	–	–	F^a^	F^a^	F^a^	F^a^
5 874 050	gCc ➔ gGc	A306G	*MSMEG_5807*	Amino acid transport	–	G	–	–	–	–	–

Genomic positions are relevant to *M. smegmatis* str. MC^2^155 (accession number NC_008596.1).

Wild‐type alleles are represented by ‘–’.

a Non‐synonymous SNP.

Bioinformatics analysis identified 19 putative SNPs compared with the laboratory *M. smegmatis* wild‐type control strains. Three SNPs were predicted to occur in pseudogenes, six SNPs were predicted to occur in intergenic regions and two SNPs were predicted as synonymous mutations. Alleles with non‐synonymous SNPs were found to occur in six *M. smegmatis* genes (Table 3) and included two genes with unknown function (*MSMEG_2746 and MSMEG_3244*), a gene predicted to be involved with homing (HNH) endonuclease function (*MSMEG_2148*), a putative 3‐dehydroquinate synthase gene (*MSMEG_3033*, *aroB*), a putative monooxygenase gene (*MSMEG_4083*) and a putative gene involved in amino acid transport (*MSMEG_5807*). It is interesting to note that more alleles with non‐synonymous SNPs were identified for the resistant mutants isolated from compound **2**, which were generated at 5× MIC compound **2** compared with 2.5× MIC compound **1**; this was due to solubility issues encountered. It was particularly interesting to find that four of the predicted SNPs occurred in the *aroB* gene, resulting in an amino acid change of G278R and C352G for all four resistant isolates to compound **2**, and two additional SNPs in *aroB*, resulting in the additional amino acid change of G280D and L359P in three out of four of these resistant isolates (Table 3). These results led us to investigate the role of this potential *aroB* target further.

The *aroB* gene from *M. smegmatis* was expressed using the *hsp60* promoter from the replicative pMV261 vector and the susceptibility of this overexpressing strain to compounds **1** and **2** was assessed using the REMA and the MICs determined. The results demonstrate that overexpression of *aroB* led to partial resistance to both compounds **1** and **2** when compared with the wild‐type strain (*t‐*test), although there is little difference between overexpression of *aroB* compared with empty vector alone (Table 4).

**Table 4:** Susceptibility profiles of *M. smegmatis* strains to compounds 1 and 2

	MIC (μg·mL^−1^)		
Strain	Compound 1	Compound 2	Rifampicin
*M. smegmatis mc* ^*2*^ *155* – wild type	56.2 ± 6.3	112.5 ± 12.5	7.5 ± 1.1
*M. smegmatis*:*aroB*	112.5 ± 12.5	300.0 ± 49.9	7.5 ± 1.1
*M. smegmatis*:pMV261	112.5 ± 12.5	275.0 ± 61.2	7.5 ± 1.1

The averages of five independent experiments are shown, and the data are presented as mean ± SEM.

## Discussion and conclusions

The piperidinol (compound **1)** and its corresponding bis‐Mannich base (compound **2)** have previously been demonstrated to have potent anti‐tubercular activity against both *M. tuberculosis* and M. bovis BCG (Jeney and Zsolnai, 1956, Abuhammad *et al.*, 2012, Abuhammad *et al.*, 2014) and, here, we have shown these compounds also have specificity for the *Mycobacterium genus* and good cytotoxicity selectivity. Despite no evident signs of cytotoxicity, the compounds appear to have a high CL_int_ rate in mouse microsomes, and hence, further chemical optimization of these compounds will be required in order to improve their metabolic degradation profile, while retaining anti‐tubercular activity.

Treatment of TB is increasingly hindered by the emergence of drug‐resistant strains and this has led to increased efforts in the identification of new potent inhibitors of *M. tuberculosis* and pharmacologically validated targets. In order to tackle this problem, there has been a recent increase in the utilisation of chemical genomics approaches to determine the cellular targets of these newly identified anti‐tubercular agents. The generation of spontaneous resistant mutants followed by whole genome sequencing has been successfully used to determine a number of intracellular targets including, for example, SQ109, an FDA approved drug to be used only for drugresistant strains of TB (Tahlan *et al.*, 2012), dinitrobenzamide and benzothiazole derivatives which are in the late stages of pre‐clinical development (Christophe *et al.*, 2009, Neres *et al.*, 2012) and the natural product pyridomycin (Hartkoorn *et al.*, 2012). From this approach a range of new and previously pharmacologically validated targets have been identified, including the MmpL3 membrane transporter (Tahlan *et al.*, 2012) that is involved in the export of trehalose monomycolate and cell wall biosynthesis, and InhA, an enoyl‐acyl carrier protein reductase that is a key component in the type II fatty acid synthase system (FasII) and is the cellular target for isoniazid (Banerjee *et al.*, 1994).

The piperidinol compound **1** inhibits the NAT enzyme from M. marinum through the generation of a polyvinyl moiety that forms a covalent adduct with the cysteine residue located in the NAT active site (Abuhammad *et al.*, 2010, Abuhammad *et al.*, 2012, Abuhammad *et al.*, 2014). However, the potential link between the anti‐tubercular activity observed for compound **1** and the potential endogenous role of NAT in the bactericidal activity of this piperidinol derivative has yet to be evaluated, and other potential targets have not been excluded (Abuhammad *et al.*, 2012, Abuhammad *et al.*, 2014). Therefore, in this study, we utilized a chemical genetic approach as a route to determine other potential cellular targets of compounds **1** and **2**. Given that *M. smegmatis* has been successfully used in high‐throughput screening campaigns to identify new TB drugs, including the clinically approved anti‐tubercular agent bedaquiline (Andries *et al.*, 2005), we chose to use this organism to generate spontaneous resistant mutants due to the ease and speed of manipulation despite the slight increase in the MIC of compounds **1** and **2** compared to *M. tuberculosis*. Interrogation of the genomes of the resultant resistant isolates has led to the identification of a multiple number of high‐quality SNPs for each mutant. The high number of SNPs observed for this piperidinol scaffold is higher than that of similar chemical bioinformatics studies (Hartkoorn *et al.*, 2012, Tahlan *et al.*, 2012, Hartkoorn *et al.*, 2014), suggesting a potentially diverse profile of the mechanisms involved..

Mycobacteria have a unique cell wall structure, comprising a mycolic acid–arabinogalactan–peptidoglycan core (Brennan and Nikaido, 1995). Investigation into the endogenous role of the NAT enzyme in mycobacteria by deletion of the *nat* gene in M. bovis BCG has demonstrated that NAT plays a key role in the biosynthesis of mycobacterial mycolic acids and this enzyme has been postulated to have a potential role in maintaining the homeostasis of the acetyl CoA pool (Bhakta *et al.*, 2004). NAT enzymes utilize the cofactor acetyl CoA as an acetyl donor for arylamine substrates (Sim *et al.*, 2007). Although, under these experimental conditions, our studies did not identify any SNPs in the *nat* gene, it was of particular interest to note the identification of a number of SNPs in the *aroB* enzyme in all resistant isolates generated with compound **2**. The *aroB* gene putatively encodes for a 3‐dehydroquinate synthase enzyme involved in the second step of the shikimate pathway with AroB catalysing the cyclization of 3‐deoxy‐d‐arabino‐heptulosonate‐7‐phosphate in 3‐dehydroquinate. The 3‐dehydroquinate is subsequently metabolized in the shikimate pathway to chorismate, which is required for the biosynthesis of all aromatic amino acids and other key metabolites. In *M. tuberculosis*, the *aroB* gene (Rv2538) is predicted to be essential for the survival of *M. tuberculosis* (Parish and Stoker, 2002, Sassetti *et al.*, 2003)*.* Enzymes within the shikimate pathway represent excellent targets for the development of new antibiotics as they are essential for bacterial survival but have no human counterpart (Bentley, 1990, Ducati *et al.*, 2007). Given the role of the AroB enzyme in the essential biosynthesis of aromatic amino acids, naphthoquinones, menaquinones and mycobactin in *M. tuberculosis*, we selected this prospective genetic target for further validation. Overexpression of *aroB* using the replicative pMV261 vector, with a relative copy number of twofold to threefold (Stover *et al.*, 1991), resulted in partial resistance of these strains to compounds **1** and **2**, which could in part be explained by the presence of the chromosomal wild‐type *aroB* gene still being expressed. Partial resistance has been found in a similar chemical bioinformatics study to investigate the mechanism of resistance to clofazimine, a drug that is used in the multidrug therapy of leprosy (Hartkoorn *et al.*, 2014), although it is possible that these studies have also identified off‐target compensatory pathways in response to these compounds. However, it is probable that given the high number of SNPs identified for this piperidinol derivative for *M. smegmatis*, these compounds are active against a diverse set of intracellular targets, which subsequently results in difficulties in restoring the observed chemical genetic phenotype through overexpression of a single gene.

In summary, these studies indicate that the cyclic piperidinol class of compound along with its bis‐Mannich base derivative is specific for the *Mycobacterium genus* and rapidly kill *M. smegmatis* with encouraging cytotoxicity profiles. Genetic interrogation of spontaneous resistant mutants of *M. smegmatis* generated under the selective pressure of compounds **1** and **2** has led to the identification of potential genetic targets and pathways that are involved in the multiple and diverse modes of action for these piperidinol chemical moieties. Previous *in vitro* biochemical studies demonstrated the formation of a polyvinyl ketone moiety, which is likely to be highly reactive at the proteomic level. Given the increase in TB drug‐resistant strains that often results from drugs acting on a single biological target, chemical moieties with anti‐tubercular inhibitory activities that act upon multiple cellular targets are highly attractive, from a clinical standpoint, for the development of new TB agents and are worthy of further investigation.

## Author contributions

C.G., E.T., G.K., M.I.G., M.J.P. and EF conceived and designed the experiments. C.G., E.T., G.K., D.P., C.F., T.B., J.H. and E.F. performed the experiments. C.G., E.T., G.K., C.G., D.P., A.M., M.I.G. and EF analysed the data. E.T., G.K. and A.D.M. analysed the bioinformatics data. C.G., G.K., A.M., D.P., M.I.G., M.J.P and E.F. contributed reagents/materials/analysis tools. C.G., E.T., A.D.M. and E.F. wrote the paper.

## Conflict of interest

The authors declare no conflicts of interest.

## Declaration of transparency and scientific rigour

This Declaration acknowledges that this paper adheres to the principles for transparent reporting and scientific rigour of preclinical research recommended by funding agencies, publishers and other organisations engaged with supporting research.

## Supporting information


**Figure S1** NMR characterisation of 4‐hydroxy‐1‐methyl‐4‐phenylpiperidin‐3‐yl)(phenyl)methanone (1) in CDCl_3_: (A) ^1^H and (B) ^13^C spectra.
**Figure S2** NMR characterisation of 3,3′‐(methylazanediyl)bis(1‐phenylpropan‐1‐one) (2) in CDCl_3_: (A) ^1^H and (B) ^13^C spectra.
**Figure S3** *M. smegmatis* checkerboard assay of compound 1 and 2 with isoniazid. Minimum inhibitory concentrations were determined for compound 1 and compound 2 with and without isoniazid at the concentrations shown. Fractional inhibitory concentrations (FICs) were determined for each compound as the MIC of the compound alone divided by the MIC of the compound in the presence of isoniazid. The sum of the FICs was used to determine the nature of the interactions.
**Figure S4** Ovine blood agglutination assay in the presence of compound 1 and compound 2. The effect of compounds 1 and 2 on the agglutination of ovine blood was determined at the concentrations indicated. The compounds were incubated with ovine blood for 1 hour. A positive agglutination control of 25 % polyethylenamine was added. After this time the microtiter plate was assessed visually for signs of agglutination. The experiment was carried out in triplicate.
**Figure S5** LC‐MS traces of the metabolic stability of compounds 1 and 2 with mouse microsomes. A) compound 1 B) compound 2 C) carbamazepine. The stability of compounds 1 and 2 (1 μg/mL) were assessed in mouse microsomes (100 μg total protein) along with carbamazepine. Samples were stopped at the time points indicated (0 – 30 mins) and analysed by LC‐MS in order to determine the amount of parent compound remaining over time.
**Table S1** Oligonucleotides used in these studies for overexpression studies in *M. smegmatis*. Restriction recognition sites are underlined.
