# Supplementary material for: Identification of the anti‐mycobacterial functional properties of piperidinol derivatives
Source: Br J Pharmacol. 2017 Mar 23;174(14):2183–93. doi: 10.1111/bph.13744 (PMC5481654; doi:10.1111/bph.13744)
Supplement: Supplementary file 1 — Figure S1 NMR characterisation of 4‐hydroxy‐1‐methyl‐4‐phenylpiperidin‐3‐yl)(phenyl)methanone (1) in CDCl3: (A) 1H and (B) 13C spectra. Figure S2 NMR characterisation of 3,3′‐(methylazanediyl)bis(1‐phenylpropan‐1‐one) (2) in CDCl3: (A) 1H and (B) 13C spectra. Figure S3 M. smegmatis checkerboard assay of compound 1 and 2 with isoniazid. Minimum inhibitory concentrations were determined for compound 1 and compound 2 with and without isoniazid at the concentrations shown. Fractional inhibitory concentrations (FICs) were determined for each compound as the MIC of the compound alone divided by the MIC of the compound in the presence of isoniazid. The sum of the FICs was used to determine the nature of the interactions. Figure S4 Ovine blood agglutination assay in the presence of compound 1 and compound 2. The effect of compounds 1 and 2 on the agglutination of ovine blood was determined at the concentrations indicated. The compounds were incubated with ovine blood for 1 hour. A positive agglutination control of 25 % polyethylenamine was added. After this time the microtiter plate was assessed visually for signs of agglutination. The experiment was carried out in triplicate. Figure S5 LC‐MS traces of the metabolic stability of compounds 1 and 2 with mouse microsomes. A) compound 1 B) compound 2 C) carbamazepine. The stability of compounds 1 and 2 (1 μg/mL) were assessed in mouse microsomes (100 μg total protein) along with carbamazepine. Samples were stopped at the time points indicated (0 – 30 mins) and analysed by LC‐MS in order to determine the amount of parent compound remaining over time. Table S1 Oligonucleotides used in these studies for overexpression studies in M. smegmatis. Restriction recognition sites are underlined. [file BPH-174-2183-s001.pdf]

Supplementary Information

Identification of the anti-mycobacterial functional properties of piperidinol derivatives

Collette S. Guy[1,2†], Esther Tichauer[1†], Gemma L. Kay[3], Daniel J. Phillips[2], Trisha L. Bailey[2], James Harrison[1], Christopher M. Furze[1], Andrew D. Millard[3], Matthew I. Gibson[2,3], Mark J. Pallen[3], Elizabeth Fullam[1*]

1) School of Life Sciences, University of Warwick, Coventry, CV4 7AL, United Kingdom

2) Department of Chemistry, University of Warwick, Coventry, CV4 7AL, United Kingdom

3) Medical School, University of Warwick, Coventry, CV4 7AL, United Kingdom

* Correspondence: Elizabeth Fullam, School of Life Sciences, University of Warwick, Coventry, CV4 7AL, UK. E-mail: e.fullam@warwick.ac.uk

Running title: piperidinol mycobacterial investigation

Key words: drug discovery, antimicrobial, target identification, chemical bioinformatics, tuberculosis, *Mycobacterium tuberculosis*

Synthesis Experimental Section

Analytical methods

NMR spectroscopy (^{1}H, ^{13}C) was conducted on a Bruker DRX-500 or Bruker AV III-600 spectrometer using deuterated chloroform as solvent and all chemical shifts (δ) are given in ppm relative to the solvent reference. Data are recorded as follows: chemical shift (multiplicity (s for singlet, d for doublet, t for triplet, m for multiplet, br for broad), integration, coupling constant(s) in Hz). High resolution mass spectra were recorded on a Bruker Electrospray Ultra-High Resolution tandem TOF mass spectrometer using electrospray ionization (ESI) in positive mode on samples prepared in methanol. FTIR spectra were acquired using a Bruker Vector 22 FTIR spectrometer with a Golden Gate diamond attenuated total reflection cell. A total of 64 scans were collected on samples in their native state.

Synthesis of 4-hydroxy-1-methyl-4-phenylpiperidin-3-yl)(phenyl)methanone 1

Methylamine hydrochloride (1.50 g, 22.22 mmol), paraformaldehyde (2.67 g, 88.89 mmol) and acetophenone (10.68 g, 10.37 mL, 88.89 mmol) were added to a 250 mL round bottomed flask containing acetonitrile (120 mL) and stirred. Concentrated hydrochloric acid (0.30 mL) was added and the mixture heated to reflux (99 °C) for 20 hours. After this time, the solution was cooled to room temperature and the solvent removed *in vacuo*. The crude mixture was dissolved in ethyl acetate (70 mL) and extracted with saturated $NaHCO_3$ (2 x 70 mL), water (1 x 50 mL) and brine (1 x 50 mL) and concentrated *in vacuo* to leave a viscous yellow oil which was purified by column chromatography on silica using 1% triethylamine in diethyl ether as eluent. Removal of the solvent left a pale yellow solid which was re-crystallised from methanol to leave compound **1** as a white solid (3.05 g, 46.5 %).

^{1}H NMR (500.133 MHz, $CDCl_3$) δ_{ppm}: 7.89 (2H, d, $J_{13\text{-}14}$ = 7.70 Hz, H^{13}); 7.57 (1H, t, $J_{15\text{-}14}$ = 7.70 Hz, H^{15}); 7.51 (2H, d, $J_{3\text{-}2}$ = 7.25 Hz, H^3); 7.44 (2H, t, $J_{14\text{-}13,\ 14\text{-}15}$ = 7.70 Hz, H^{14}); 7.26 (2H, t, $J_{2\text{-}1,\ 2\text{-}3}$ = 7.25 Hz, H^2); 7.15 (1H, t, $J_{1\text{-}2}$ = 7.25 Hz, H^1); 5.17 (1H, d, $J_{16\text{-}6}$ = 2.85 Hz, H^{16}); 4.43 (1H, dd, $J_{10\text{-}9}$ = 11.65 Hz, 3.80 Hz, H^{10}); 2.95 (1H, dd, $J_{9\text{-}10}$ = 11.65 Hz, $J_{9\text{-}9}$ = 3.80 Hz, H^9); 2.81 (1H, m, H^7); 2.71 (2H, m, H^7, H^9); 2.42 (3H, s, H^8); 2.09 (1H, m, H^6); 1.84 (1H, dt, $J_{6\text{-}6}$ = 14.20 Hz, $J_{6\text{-}7}$ = 2.85 Hz, H^6).**^{13}C NMR** (125.773 MHz, $CDCl_3$) δ_{ppm}: 204.3 (C^{11}); 147.1 (C^4); 135.9 (C^{12}); 133.9 (C^{15}); 128.8 (C^{14}); 128.3 (2C, C^2, C^{13}); 126.8 (C^1); 124.8 (C^3); 72.6 (C^5); 54.7 (C^9); 51.4 (C^7); 50.6 (C^{10}); 46.0 (C^8); 40.0 (C^6). **IR** cm^{-1}: 3421 (O-H stretch); 2970, 2938 (alkyl C-H stretch); 2801 ($R_2N\text{-}CH_3$ stretch); 1661 (C=O stretch); 1596, 1589, 1446 (C=C vibrations).**MS** (ESI +) m/z: 296.1643 $[M+H]^+$; expected 296.1645 ($C_{19}H_{22}NO_2$).

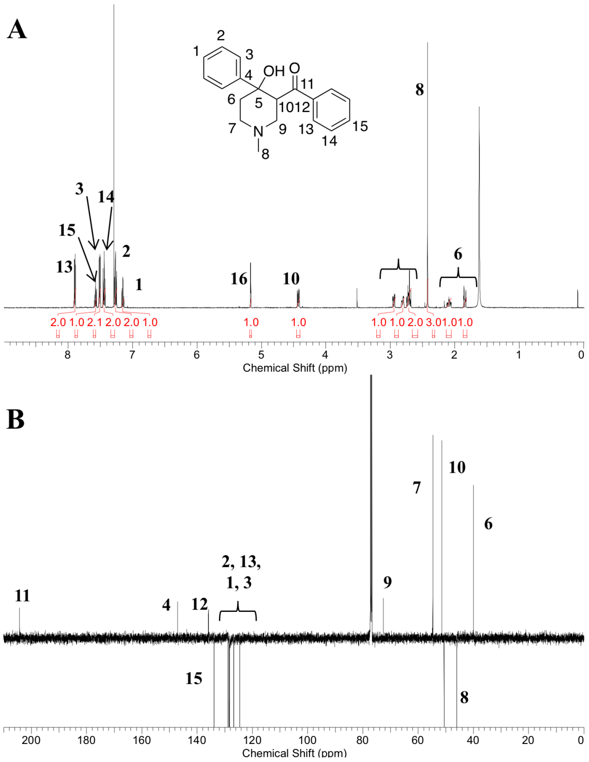

Supplementary Figure S1 NMR characterisation of 4-hydroxy-1-methyl-4-phenylpiperidin-3-yl)(phenyl)methanone **(1)** in $CDCl_3$: (A) ^{1}H and (B) ^{13}C spectra.

Synthesis of 3,3'-(methylazanediyl)bis(1-phenylpropan-1-one) 2

Methylamine hydrochloride (1.50 g, 22.22 mmol), paraformaldehyde (2.67 g, 88.89 mmol) and acetophenone (10.68 g, 10.37 mL, 88.89 mmol) were added to a 250 mL round bottomed flask containing acetonitrile (120 mL) and stirred. Concentrated hydrochloric acid (0.30 mL) was added and the mixture heated to reflux (99 °C) for 20 hours. After this time, the solution was cooled to room temperature and the solvent removed *in vacuo*. The crude mixture was dissolved in ethyl acetate (70 mL) and extracted with saturated $NaHCO_3$ (2 x 70 mL), water (1 x 50 mL) and brine (1 x 50 mL). The organic layer was further dried over magnesium sulfate and the solvent removed *in vacuo* to leave a viscous yellow oil. Upon the addition of cool ethyl acetate (30 mL), a white precipitate formed which was collected by filtration and washed thoroughly with ethyl acetate to leave a white powder (compound **2**, 0.95 g).

^{1}H NMR (500.133 MHz, $CDCl_3$) δ_{ppm}: 8.01 (4H, d, $J_{3\text{-}2}$ = 7.38 Hz, H^3); 7.62 (2H, t, $J_{1\text{-}2}$ = 7.38 Hz, H^1); 7.49 (4H, t, $J_{2\text{-}1,\ 2\text{-}3}$ = 7.38 Hz, H^2); 3.79 (4H, t, $J_{6\text{-}7}$ = 6.80 Hz, H^6); 3.56 (4H, t, $J_{7\text{-}6}$ = 6.80 Hz, H^7); 2.81 (3H, s, H^8). **^{13}C NMR** (125.773 MHz, $CDCl_3$) δ_{ppm}: 195.8 (C^5); 135.5 (C^4); 134.2 (C^3); 128.9 (C^2); 128.3 (C^1); 51.9 (C^7); 40.7 (C^8); 33.6 (C^6). **IR** cm^{-1}: 3050 (C=C-H stretch); 2916 (alkyl C-H stretch); 1676 (C=O stretch); 1596, 1581, 1479 (C=C vibrations). **MS** (ESI +) m/z: 296.1 $[M+H]^+$.

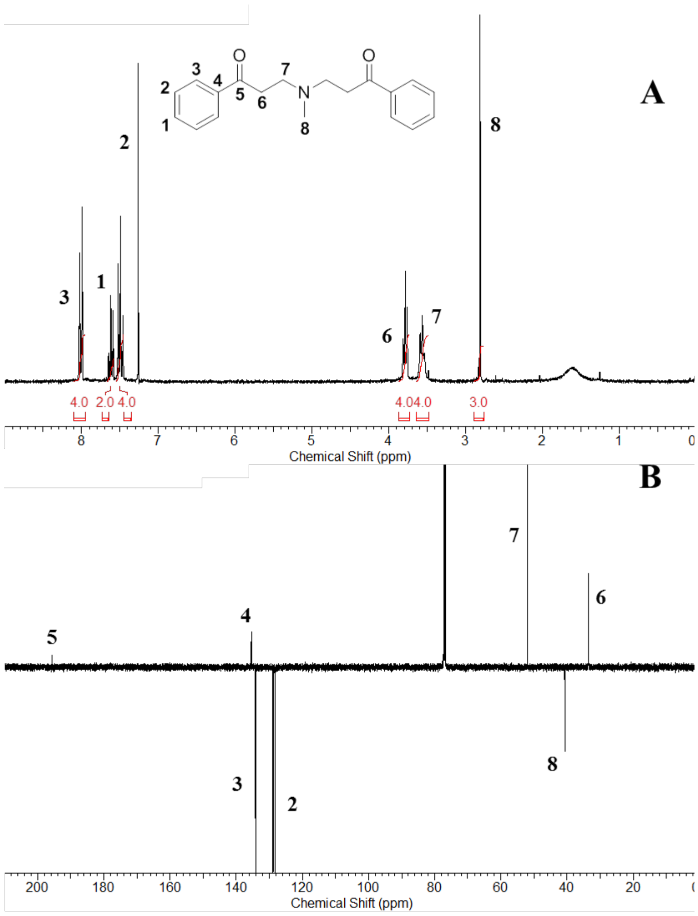

Supplementary Figure S2: NMR characterisation of 3,3'-(methylazanediyl)bis(1-phenylpropan-1-one) **(2)** in $CDCl_3$: (A) ^{1}H and (B) ^{13}C spectra.

Supplementary Figure 3

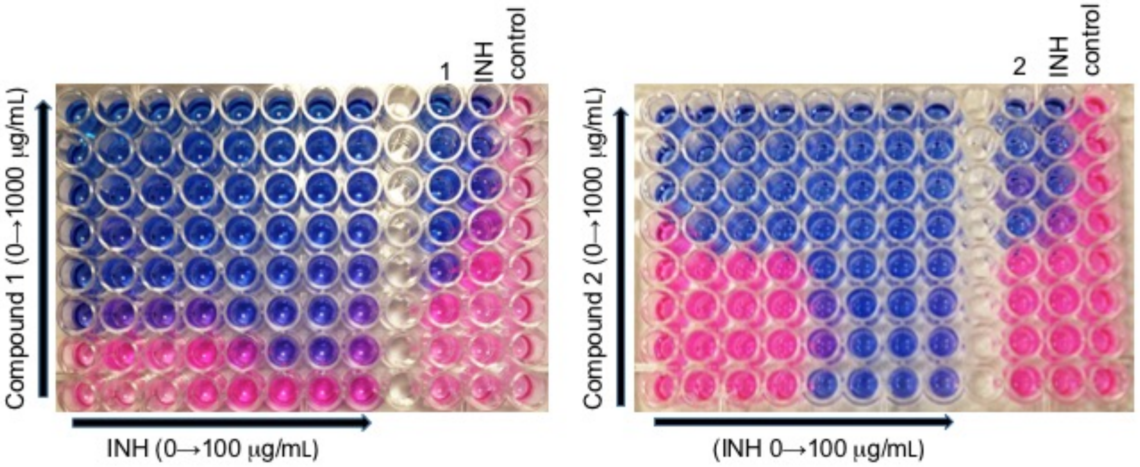

Supplementary Figure 3: *Mycobacterium* s*megmatis* checkerboard assay of compound 1 and 2 with isoniazid. Minimum inhibitory concentrations were determined for compound **1** and compound **2** with and without isoniazid at the concentrations shown. Fractional inhibitory concentrations (FICs) were determined for each compound as the MIC of the compound alone divided by the MIC of the compound in the presence of isoniazid. The sum of the FICs was used to determine the nature of the interactions.

Supplementary Figure 4

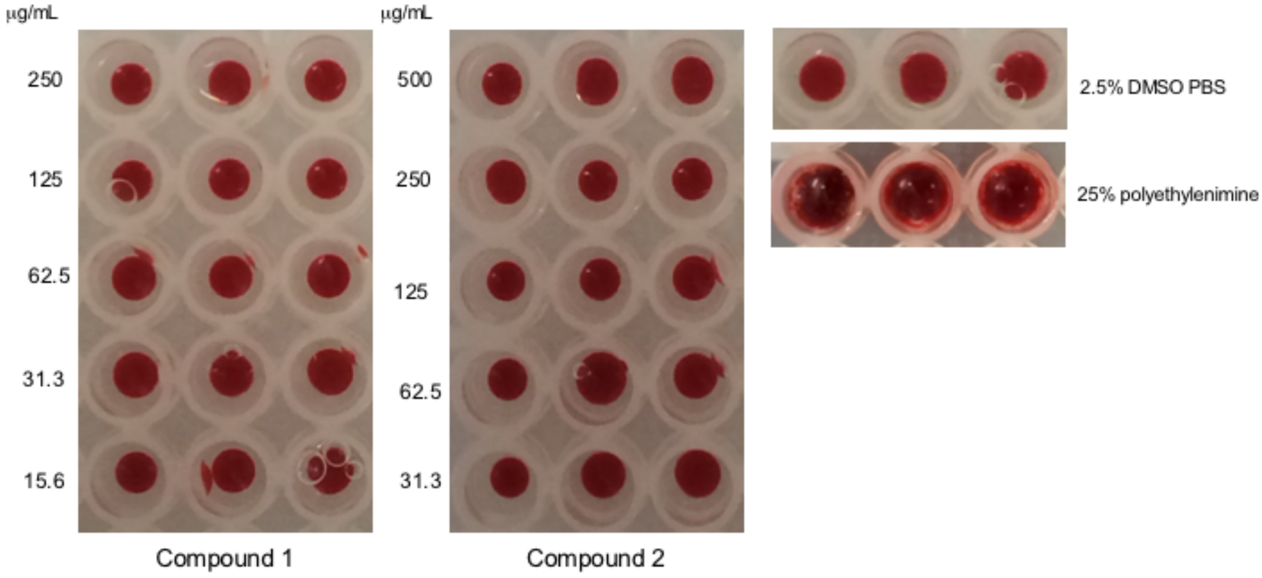

Supplementary Figure 4. Ovine blood agglutination assay in the presence of compound 1 and compound 2. The effect of compounds **1** and **2** on the agglutination of ovine blood was determined at the concentrations indicated. The compounds were incubated with ovine blood for 1 hour. A positive agglutination control of 25 % polyethylenamine was added. After this time the microtiter plate was assessed visually for signs of agglutination. The experiment was carried out in triplicate.

Supplementary Figure 5

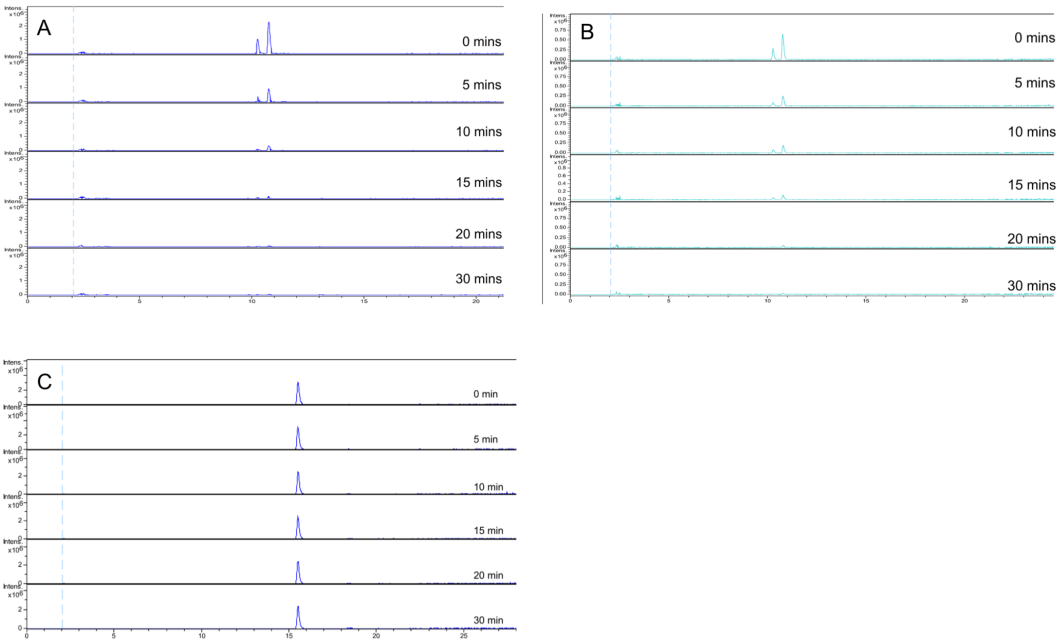

Supplementary Figure 5: LC-MS traces of the metabolic stability of compounds 1 and 2 with mouse microsomes. A) compound **1** B) compound **2** C) carbamazepine. The stability of compounds **1** and **2** (1 μg/mL) were assessed in mouse microsomes (100 μg total protein) along with carbamazepine. Samples were stopped at the time points indicated (0 – 30 mins) and analysed by LC-MS in order to determine the amount of parent compound remaining over time.

Supplementary Table 1: Oligonucleotides used in these studies for overexpression studies in *M. smegmatis*: Restriction recognition sites are underlined:

**Name**	**Sequence (5'-3')**
aroB_F	AAAA<u>GGATCC</u>AGTGATCGTCGAGGTGAAGGTGG
aroB_R	AAAA<u>AAGCTT</u>TCAGAGCTGATCCGGGCTCAGC
pMV261_F	CGAGTGGCAGCGAGGACAAC
pMV261_R	TGTGCAATGTAACATCAGAG
